# Early Detection of Maternal Risk for Preeclampsia

**DOI:** 10.5402/2012/172808

**Published:** 2012-07-17

**Authors:** B. Mikat, A. Gellhaus, N. Wagner, C. Birdir, R. Kimmig, A. Köninger

**Affiliations:** ^1^Department of Obstetrics and Gynecology, University Hospital Essen, Hufelandstraße 55, 45147 Essen, Germany; ^2^Institute of Molecular Biology, University Hospital, Hufelandstraße 55, 45147 Essen, Germany; ^3^Harris Birthright Research Centre for Fetal Medicine, King's College Hospital, London SE5 9RS, UK

## Abstract

Preeclampsia is one of the leading causes of maternal and fetal morbidity and mortality. New molecular insights offer new possibilities of early diagnosis of elevated maternal risk. Maternal risk factors, biophysical parameters like Doppler examination of the uterine arteries and biochemical parameters allow early risk calculation. Preventive and effective therapeutic agents like acetylsalicylacid can be started in the early second trimester. This article reviews the diagnostic possibilities of early risk calculation to detect women having high risk for preeclampsia and the potential benefits for them, the offspring and health care systems. We provide risk calculation for preeclampsia as an important and sensible part of first trimester screening.

## 1. Introduction

2–7% of pregnant women are affected from preeclampsia (PE) [1] which occurred in the second half of pregnancy and is defined mainly by the symptoms hypertension and proteinuria. 5–8% of these women develop HELLP syndrome (hemolysis, elevated liver enzymes, and low platelet count). Preeclampsia is one of the leading causes of maternal and fetal mortality worldwide and a main cause of preterm labour. Women with a history of preeclampsia are at elevated risk for cardiovascular diseases later in life [2]. To reduce morbidity and mortality resulting from this disease life-style changes and prevention should be the main aim. The early detection for a high risk to develop preeclampsia has the potential to be a predictive tool also for other health disorders with meaningful consequences for the mothers, their offspring, and health care systems.

## 2. Pathogenesis

The placenta plays a key role in the pathogenesis of preeclampsia since the symptoms of PE can occur in molar pregnancies which lack a fetus and the disease disappeared once the placenta is delivered.

Impaired placental function leads to fetal malnutrition and deprivation of oxygen and nutrition supply. It is meanwhile well accepted that undernutrition results in intrauterine growth restriction which seems to program coronary heart disease and hypertension later in life [3–5]. Strikingly the human placenta is only a transient organ, but its effect on the offspring is conserved throughout life. Appropriate function of the placenta requires the correct differentiation of the trophectoderm to establish a nutrition route between embryo and mother [6]. Despite many years of research, a complete understanding of the molecular pathogenesis of PE is still missing. 

The current theory of the pathogenesis of PE as reviewed by Christopher Redman and Ian Sargent is thought to occur as a 2-stage process with poor placentation in the first half of pregnancy resulting in the maternal response in the second half of pregnancy [7, 8]. Anatomic placental examination reveals that the basal plate is most affected by this disease, the site where cytotrophoblast (CTB) invasion occurs [9]. In PE, interstitial CTB invasion and endovascular invasion are shallow and thus adequate vascular remodeling of the spiral arteries is impaired [10]. The second stage of PE is thought to be the maternal response to abnormal placentation resulting from endothelial dysfunction and an imbalance in circulating angiogenic/vasculogenic factors such as soluble vascular endothelial growth factor receptor-1 (VEGFR-1, sFlt-1), placental growth factor (PlGF), and the transforming growth factor-beta receptor endoglin (CD105) (reviewed by [9, 11]). In 2011 the role of angiogenic proteins in developing preeclampsia was strengthened since these data will improve the detection and treatment of preeclampsia.

### 2.1. New Molecular Insights

There is mounting evidence that a nonphysiological hypoxic environment later in pregnancy may result in this deregulation of angiogenic factors at the maternal-fetal interface. Recently it has been shown that early preeclampsia is associated with abnormalities in oxygen sensing since early preeclamptic placentas are unable to regulate HIF1- (hypoxia-inducible factor 1-) alpha levels [12]. Chronic exposure to nonphysiological oxygen levels in preeclampsia decreases VEGF (vascular endothelial growth factor) whereas sFlt-1 is highly upregulated. It is well known that secreted sFlt-1 binds to VEGF and PlGF with high affinity and thereby decreasing their ability to bind to their receptors [13]. These changes act like an antiangiogenic therapy which has been shown in clinical trials leading to similar clinical symptoms such as impaired angiogenesis especially maturation of vessels, hypertension, proteinuria and edema [14, 15]. Verlohren et al. [16] reported that the sFlt-1/PlGF ratio is important to identify women at risk for delivery and is a reliable tool to discriminate between different types of pregnancy-related hypertensive disorders. In women with suspected preeclampsia at <34 weeks, the circulating sFlt1/PlGF ratio predicts adverse outcomes occurring within two weeks [17, 18]. However, the mechanisms by which placenta-derived sFlt1 gains access to the maternal circulation remain unclear. Rajakumar and colleagues [19] report that the sFlt1 protein is highly enriched in syncytial knots which easily detach from the syncytiotrophoblast—a finding which is increased in preeclampsia. These multinucleated aggregates are metabolically active and are capable of de novo synthesis and may thus contribute to the maternal vascular injury in PE [19].

Moreover we revealed a deregulated expression of another molecule found in the bulk of changed molecules in PE, the matricellular CCN3 protein which lead to an imbalance in proliferation and migration of human trophoblast cells and could contribute to the shallow invasion of trophoblast cells into the decidual compartment and spiral arteries observed in preeclampsia [20–23]. In addition, in our recent publication we could show that the cholesterol transporter ABCA1 is deregulated in early-onset preeclampsia resulted from placental hypoxia [24, 25]. These results focused on the importance of the maternal-fetal cholesterol transport for adequate development of the fetus. 

Microarray datasets of basal plate biopsies of both normal placentation and PE (24–36 weeks) demonstrated novel observations indicating increased expression of the leptin receptor Siglec-6 and pappalysin (PAPP-A2), a metalloproteinase that cleaves insulin-like growth factor (IGF) binding protein-5 (IGFBP-5), in PE placentas compared to controls [9]. Overall these results suggest alterations in important biological processes including pathways that are regulated by leptin and IGF signals. 

## 3. Early Diagnosis

The aim for the early diagnosis is to start a preventive therapy by administration of 100 mg acetylsalicylacid (ASS, aspirin) before 16 weeks of pregnancy (reduction of risk for severe preeclampsia: RR 0, 1; 95% KI 0, 1–0, 74) [26]. It is clear that a risk calculation in first trimester would be the most effective method to prevent preeclampsia.

Since the data on the usefulness of early administration of aspirin is still emerging, the optimal dose, which is probably 70–160 mg/d, is still under investigation. There is a known aspirin resistance in 33% of all women, which justifies the introduction of at least 100 instead of 80 mg aspirin/d. The combination of aspirin and low-molecular-weight heparin in secondary prevention seems to bring an additional benefit over aspirin alone [27], especially for an additional hereditary thrombophilia [28].

Early detection is based on three main points which are focused on and complement each other: a detailed medical history, the collection of biophysical parameters such as blood pressure, arterial stiffness, and Doppler examination of maternal blood vessels, and the determination of biochemical parameters, which can give clues to impaired placental function.

## 4. Maternal Risk Factors

The risk factors that are involved in the development of preeclampsia are also the symptoms of the metabolic syndrome and glucose metabolism disorders such as diabetes mellitus as well as insulin resistance and assisted reproductive techniques, increased body mass index (>35 kg/m²) and elevated diastolic blood pressure >80 mm Hg [29]. Further risk factors are positive family history of preeclampsia, multiple pregnancy, pregnant women over 40 years, preexisting renal disease, and clotting disorders [30, 31].

Particularly common clotting disorders associated with an increased risk for preeclampsia are factor V Leiden mutation, homozygous MTHFR mutation, hyperhomocysteinemia, presence of antiphospholipid antibodies, and the combination of multiple thrombophilias [32].

Immunological causes can be attributed to the increased risk, for example, the first pregnancy with a partner. In contrast, multiparity with the same partner reduces the risk [33].

Only regarding history, 30% of women with PE are detected early with a false positive rate of 5% [29]. Regarding the pregnancy-induced hypertension without preeclampsia, the maternal history is of a much greater importance than the serum parameters and the pulsatility index of the uterine arteries [34].

## 5. Biophysical Parameters

Mean arterial blood pressure in the first trimester can be used in combination with maternal risk factors as a predictive marker of PE in the first trimester which has a detection rate of 76% for early-onset PE. Systolic blood pressure is already significantly different in the first trimester in view of the early- and late-onset PE and pregnancy-induced hypertension [35].

The arterial supply to the uterus occurs mostly through uterine arteries, which turn into circular running arteriae arcuatae. Here radial arteries branches, the spiral arteries, penetrate deeply into the myometrium and supply the decidua and fetus during pregnancy.

Abnormal placentation and incomplete cytotrophoblast invasion characterized by inadequate formation and vasodilation of the spiral arteries have long been known as one of the main risk factors for development of preeclampsia [36, 37].

Based on these morphological changes, an abnormal uteroplacental circulation is typically characterized by a persistence of the postsystolic (Notch) and high resistance indices.

A prediction of the severe form of pregnancy-induced hypertension and preeclampsia is possible by examining the uteroplacental vessels in the first and second trimesters.

Various publications showed that in first trimester screening, Doppler examination of the uterine arteries identified a certain percentage of pregnant women that later develop preeclampsia with elevated uterine resistance indices and postsystolic incisions [38–40].

About 40% of pregnant women can thus be detected at a false-positive rate of 5% [34, 41]. However, the sensitivity for the prediction of preeclampsia is significantly lower than that in second-trimester ultrasound measurements.

Higher rates of sensitivity regarding the discovery of a late onset preeclampsia can be achieved in the second trimester of pregnancy. Several Doppler studies in second trimester yielded detection rates of 70–80% [42, 43].

## 6. Biochemical Parameters

The problem of the Doppler examination alone, however, lies in the low predictive value. Only in combination with biochemical markers, this evaluation is clinically relevant for a preventive therapy.

In the second trimester, the combination of Doppler sonography and angiogenic factors such as PlGF/sEndoglin (sEng) and sFlt-1 is a valid prediction of preeclampsia [44].

In order to intervene preventively, high risk population should be identified before the 16th week of pregnancy. The aim is, therefore, to predict preeclampsia at first trimester of pregnancy.

PAPP-A was first identified as a predictive marker (see below, [34]). PlGF is also in the first quarter of pregnancy decreased (see below). Further promising targets for first trimester screening are PP-13, soluble endoglin, inhibin A, activin A, pentraxin 3, P-selectin, IGFBP-1 and 3, adiponectin, resistin, L-arginine, asymmetric dimethylarginine (ADMA), and homoarginine. However, sFlt-1 is not suitable for screening in the first trimester.

The aim of scientific papers on the subject of preeclampsia is to develop a test that can predict preeclampsia in the first trimester of pregnancy and can be applied in clinical routine. In the following section, some markers are shown which are in focus of the current research. The discussion about the clinical usefulness will be discussed below.

### 6.1. PIGF (Placental Growth Factor)

PlGF belongs to the VEGF family, is secreted by trophoblast cells, and has proangiogenic function. Preeclampsia occurs due to an impaired placentation with subsequent ischemia resulting in an increased secretion of antiangiogenic factors such as sFlt-1 (soluble Fms-like tyrosine kinase-1) and sEng (soluble endoglin) in the maternal circulation. This process leads to a course of antagonizing the angiogenic factors such as PlGF [45].

PIGF was in an early focus of the research groups in the search for a suitable prediction factor. It turned out that the concentration of PlGF in a preeclamptic pregnancy did not increase to the extent as would be expected in a normal pregnancy, as shown by us [46, 47]. Others could show that in first trimester, there are already significant differences between PlGF concentrations in maternal blood of pregnant women with normal pregnancy and those that develop preeclampsia during pregnancy [34, 48–50]. Since 2011, the first conventional test of the company Alere allows the quantitative detection of PIGF in anticoagulated EDTA plasma in the first trimester with fluorescence immunoassay (sensitivity and specificity 95%). The detection rate of preeclampsia using PlGF alone for the early-onset preeclampsia is between 41% and 59% and for late-onset preeclampsia 33% [51].

### 6.2. sFlt/PlGF Ratio

Research on anti-angiogenesis factors such as sFlt-1 failed to convince as the exclusive marker for the prediction of preeclampsia in the first trimester [51]. Verlohren et al. showed that the combination of angiogenesis and antiangiogenesis factors, at least in the second and third trimesters, may offer the possibility of a risk classification by an sFlt/PlGF ratio. It was found that patients with preeclampsia had a significantly increased sFlt/PlGF ratio compared to patients with a normal pregnancy [16].

### 6.3. PAPP-A

PAPP-A (pregnancy-associated plasma protein A), an insulin-like growth factor binding protein protease, is secreted by the syncytiotrophoblast. As part of the first-trimester screening, it has long been used in risk calculation for chromosomal abnormalities. We could show that patients with decreased levels of PAPP-A in maternal blood during the first trimester develop preeclampsia [52], especially an early-onset preeclampsia as revealed also by others [34, 53, 54].

### 6.4. Inhibin A and Activin A

Both glycoprotein hormones are produced by the fetoplacental unit. Several studies exhibited that both inhibin A and activin A are increased in the first trimester in maternal blood of patients who later develop preeclampsia compared to pregnant women with normal pregnancies [55, 56]. However, no association is found between impaired trophoblast invasion and subsequent endothelial dysfunction and increased concentration of activin A [56].

### 6.5. PP13

The placental protein 13 plays a role in the physiological placentation. Because of impaired placentation in the presence of preeclampsia, there is an increased secretion of PP13 in the first trimester of pregnancy [57–61].

### 6.6. PTX3

Pentraxin 3 is a secreted protein as part of an inflammatory immune response and is increased as an acute phase protein molecule [62]. Both with manifestations of PE as well before clinical symptoms, there is an increased secretion of PTX 3 in the maternal circulation [54, 63, 64].

### 6.7. P-Selectin

As a cell adhesion molecule, P-selectin plays a role in endothelial dysfunction. The consequence of placental ischemia in the context of preeclampsia is endothelial dysfunction and thus increased secretion of P-Selectin [65]. This is already detectable in the first trimester of pregnancy [54, 63, 64].

### 6.8. IGFBP-1 and 3

Both insulin-like growth factor binding proteins are the focus of new research. Both, in early- and late- onset preeclampsia, IGFBP-1 is decreased in the first trimester. Such changes are detected by secretion of IGFBP-3 only in late-onset preeclampsia. In both cases, there is no correlation to a disturbed trophoblast invasion [66, 67].

### 6.9. Adiponectin

In the case of early-onset PE, adiponectin levels are higher than in the first trimester compared to normal controls. This does not apply to late-onset PE. There is no relationship between adiponectin and PAPP-A levels and Doppler values. In addition, there is no advantage in prediction by the addition of adiponectin [68].

### 6.10. Resistin

Resistin levels in the first trimester are higher in patients who develop preeclampsia than controls. There is no relationship to impaired placental perfusion [69].

### 6.11. L-Arginine, Asymmetric Dimethylarginine (ADMA), and Homoarginine

All three substances are part of NO metabolism. L-arginine and L-homoarginine are increased in the first trimester at later-developing early-onset preeclampsia, as well as the ratio of ADMA /L-arginine and ADMA /L-homoarginine. This is not the case for late-onset preeclampsia and for the isolated analysis of ADMA [70].

## 7. Outlook

All biophysical and biochemical markers shown are used for prediction of preeclampsia. 

Meanwhile it is clear that a single diagnostic marker does not have the strength to safely predict subsequent preeclampsia. For this reason, it seems to be promising to use history, biophysical, and several biochemical parameters to ensure the best possible detection rate achieved.

Finally, one must distinguish between early- and late- onset preeclampsia in order to classify the present results correctly.

The early-onset preeclampsia is defined as the onset before 34 weeks of pregnancy, the intermediate-onset preeclampsia between the 34 and 37 weeks and the late-onset preeclampsia after 37 weeks. The late-onset PE seems to follow a different pathogenetic mechanism, since the serum parameters differ significantly as a marker of disturbed placentation in terms of predictive power [34]. The placentation disorder, according to previous data, is a feature of early preeclampsia. The addition of biochemical markers in the first trimester is therefore particularly suitable for detection of early preeclampsia.

Poon et al. pioneered the evaluation of a few serum parameters and maternal factors in order to achieve a good predictive power of early preeclampsia. The detection rate of early-onset PE is 93.1% in the first trimester by an algorithms from maternal risk factors, mean arterial blood pressure, pulsatility index of the uterine arteries, PAPP-A, and PlGF [34]. The detection rate for the late-onset PE with an appropriate algorithm is 44.9%.

These named parameters can now be purchased commercially and combined with appropriate software.

Akolekar et al. in 2011 found that the detection rate of preeclampsia in the first trimester by a combination of several markers (PIGF, PAPP-A, PP13, inhibin A, activin A, sEndoglin, PTX3, P-selectin, blood pressure, Dopplersonography, and history) is increased significantly to a detection rate of 91% at a fixed 5% false-positive rate for early-onset PE, 79.4% for intermediate-onset PE (34th–37th weeks of gestation), and 60% for late-onset PE [54]. The addition of these parameters allows a better predictive power of all forms of preeclampsia compared to the above-described relatively simple algorithm, having particular effect on a high detection rate for early-onset preeclampsia.

Further studies are expected, that show which of the biochemical markers are really useful in clinical practice. The relation of costs and benefit must be explored. 

Finally, the question arises that how far it may succeed in establishing the first-trimester screening tests with the consecutive possible prevention by aspirin and/or low-molecular-weight heparin, as a screening in a large, unselected collective. Since prevention is simple and inexpensive, the obstacle is much more on a personal and cost intensive screening tool. The investigation regarding chromosome abnormalities will depend on the basis of the consequences of abnormal test results of many factors and is always carried out only in a preselected collective. Screening for preeclampsia should be for a much larger collective of pregnant women, not at least because of the higher risk to get preeclampsia as a chromosomal abnormal baby and the ease of prophylaxis. Another important reason for early preeclampsia risk calculation is the fact that women with preeclampsia have a higher life-time risk for getting cardiovascular disease. Better observation of this collective of patients, changing of life-style factors, and health education could be an important step to reduce morbidity and mortality according to cardiovascular problems worldwide.

Currently the aspect of fetal programming is in the main focus of research. Not only the mother, also the offspring bear the consequences of preeclamptic pregnancy with mostly intrauterine growth restriction like elevated risk for cardiovascular diseases and behavioural disorders, for example. 

It would be desirable in the future to integrate preeclampsia risk calculation to the regular prenatal care in first trimester. Further studies on large collectives have to determine to what extent the false-positive and false-negative findings can lead in relation to health and economic disadvantages. Even an early screening should not replace careful pregnancy monitoring.

Finally, pregnancy is not only a short time in a woman's life with the aim to deliver a baby but it is also an important time giving insights in a women's health status. As we already know pregnancy may positively influence women's health future as could be shown by studies which detected a reduced risk of developing breast cancer after pregnancy [71]. As an indicator of risk factors, pregnancy is not only the beginning of taking care for a family, but also for a better self-care.

## References

[1] Villar K, Critchley H, MacLean A, Poston L (2003). Eclampsia and pre-eclampsia: a health problem for 2000 years. *Preeclampsia*.

[2] Noris M, Perico N, Remuzzi G (2005). Mechanisms of disease: pre-eclampsia. *Nature Clinical Practice Nephrology*.

[3] Barker DJP, Osmond C, Forsen TJ, Kajantie E, Eriksson JG (2007). Maternal and social origins of hypertension. *Hypertension*.

[4] Barker DJP (1995). Intrauterine programming of adult disease. *Molecular Medicine Today*.

[5] Godfrey KM, Inskip HM, Hanson MA (2011). The long-term effects of prenatal development on growth and metabolism. *Seminars in Reproductive Medicine*.

[6] Cross JC, Baczyk D, Dobric N (2003). Genes, development and evolution of the placenta. *Placenta*.

[7] Roberts JM, Cooper DW (2001). Pathogenesis and genetics of pre-eclampsia. *The Lancet*.

[8] Redman CW, Sargent IL (2005). Latest advances in understanding preeclampsia. *Science*.

[9] Winn VD, Gormley M, Fisher SJ (2011). The impact of preeclampsia on gene expression at the maternal-fetal interface. *Pregnancy Hypertension*.

[10] Zhou Y, Damsky CH, Chiu K, Roberts JM, Fisher SJ (1993). Preeclampsia is associated with abnormal expression of adhesion molecules by invasive cytotrophoblasts. *Journal of Clinical Investigation*.

[11] Kusanovic JP, Romero R, Chaiworapongsa T (2009). A prospective cohort study of the value of maternal plasma concentrations of angiogenic and anti-angiogenic factors in early pregnancy and midtrimester in the identification of patients destined to develop preeclampsia Prediction of preeclampsia. *Journal of Maternal-Fetal and Neonatal Medicine*.

[12] Rolfo A, Many A, Racano A (2010). Abnormalities in oxygen sensing define early and late onset preeclampsia as distinct pathologies. *PLoS ONE*.

[13] Banks RE, Forbes MA, Searles J (1998). Evidence for the existence of a novel pregnancy-associated soluble variant of the vascular endothelial growth factor receptor, Flt-1. *Molecular Human Reproduction*.

[14] Veronese ML, Mosenkis A, Flaherty KT (2006). Mechanisms of hypertension associated with BAY 43-9006. *Journal of Clinical Oncology*.

[15] Verheul HMW, Pinedo HM (2007). Possible molecular mechanisms involved in the toxicity of angiogenesis inhibition. *Nature Reviews Cancer*.

[16] Verlohren S, Herraiz I,  Lapaire O (2012). The sFlt-1/PlGF ratio in different types of hypertensive pregnancy disorders and its prognostic potential in preeclamptic patients. *American Journal of Obstetrics & Gynecology*.

[17] Rana S, Powe CE, Salahuddin S (2012). Angiogenic factors and the risk of adverse outcomes in women with suspected preeclampsia. *Circulation*.

[18] Perni U, Sison C, Sharma V (2012). Angiogenic factors in superimposed preeclampsia: a longitudinal study of women with chronic hypertension during pregnancy. *Hypertension*.

[19] Rajakumar A, Cerdeira AS, Rana S (2012). Transcriptionally active syncytial aggregates in the maternal circulation may contribute to circulating soluble fms-like tyrosine kinase 1 in preeclampsia. *Hypertension*.

[20] Gellhaus A, Schmidt M, Dunk C, Lye SJ, Kimmig R, Winterhager E (2006). Decreased expression of the angiogenic regulators CYR61 (CCN1) and NOV (CCN3) in human placenta is associated with pre-eclampsia. *Molecular Human Reproduction*.

[21] Gellhaus A, Schmidt M, Dunk C, Lye SJ, Winterhager E (2007). The circulating proangiogenic factors CYR61 (CCN1) and NOV (CCN3) are significantly decreased in placentae and sera of preeclamptic patients. *Reproductive Sciences*.

[22] Wolf N, Yang W, Dunk CE (2010). Regulation of the matricellular proteins CYR61 (CCN1) and NOV (CCN3) by hypoxia-inducible factor-1*α* and transforming-growth factor-*β*3 in the human trophoblast. *Endocrinology*.

[23] Yang W, Wagener J, Gellhaus A (2011). Impact of CCN3 (NOV) glycosylation on migration/invasion properties and cell growth of the choriocarcinoma cell line Jeg3. *Human Reproduction*.

[24] Plösch T, Gellhaus A, van Straten EM (2010). The liver X receptor, (LXR) and its target gene ABCA1 are regulated upon low oxygen in human trophoblast cells: a reason for alterations in preeclampsia?. *Placenta*.

[25] Ugocsai P, Hohenstatt A, Paragh G (2010). HIF-1*β* determines ABCA1 expression under hypoxia in human macrophages. *International Journal of Biochemistry and Cell Biology*.

[26] Bujold E, Morency AM, Roberge S, Lacasse Y, Forest JC, Giguère Y (2009). Acetylsalicylic acid for the prevention of preeclampsia and intra-uterine growth restriction in women with abnormal uterine artery Doppler: a systematic review and meta-analysis. *Journal of Obstetrics and Gynaecology Canada*.

[27] Gris JC, Chauleur C, Molinari N (2011). Addition of enoxaparin to aspirin for the secondary prevention of placental vascular complications in women with severe pre-eclampsia. The pilot randomized controlled NOH-PE trial. *Thrombosis and Haemostasis*.

[28] de Vries JI, van Pampus MG, Hague WM, Bezemer PD, Joosten JH, FRUIT investigators (2012). Low-molecular-weight heparin added to aspirin in the prevention of recurrent early-onset preeclampsia in women with inheritable thrombophilia: the FRUIT-RCT. *Journal of Thrombosis and Haemostasis*.

[29] Yu CKH, Smith GCS, Papageorghiou AT, Cacho AM, Nicolaides KH (2005). An integrated model for the prediction of preeclampsia using maternal factors and uterine artery Doppler velocimetry in unselected low-risk women. *American Journal of Obstetrics and Gynecology*.

[30] Sibai B, Dekker G, Kupferminc M (2005). Pre-eclampsia. *The Lancet*.

[31] Steegers EAP, von Dadelszen P, Duvekot JJ, Pijnenborg R (2010). Pre-eclampsia. *The Lancet*.

[32] Benedetto C, Marozio L, Tavella AM, Salton L, Grivon S, Di Giampaolo F (2010). Coagulation disorders in pregnancy: acquired and inherited thrombophilias. *Annals of the New York Academy of Sciences*.

[33] Skjaerven R, Wilcox AJ, Lie RT (2002). The interval between pregnancies and the risk of preeclampsia. *The New England Journal of Medicine*.

[34] Poon LCY, Staboulidou I, Maiz N, Plasencia W, Nicolaides KH (2009). Hypertensive disorders in pregnancy: screening by uterine artery Doppler at 11–13 weeks. *Ultrasound in Obstetrics and Gynecology*.

[35] Poon LCY, Kametas NA, Valencia C, Chelemen T, Nicolaides KH (2011). Hypertensive disorders in pregnancy: screening by systolic diastolic and mean arterial pressure at 11–13 weeks. *Hypertension in Pregnancy*.

[36] Brosens IA, Robertson WB, Dixon HG, Wynn RM (1972). The role of spiral arteries in the pathogenesis of preeclampsia. *Obstetrics and Gynecology Annual*.

[37] Pijnenborg R, Vercruysse L, Hanssens M (2006). The uterine spiral arteries in human pregnancy: facts and controversies. *Placenta*.

[38] Lovgren TR, Dugoff L, Galan HL (2010). Uterine artery Doppler and prediction of preeclampsia. *Clinical Obstetrics and Gynecology*.

[39] Bahado-Singh RO, Jodicke C (2010). Uterine artery Doppler in first-trimester pregnancy screening. *Clinical Obstetrics and Gynecology*.

[40] Carbillon L (2012). First trimester uterine artery Doppler for the prediction of preeclampsia and foetal growth restriction. *Journal of Maternal-Fetal and Neonatal Medicine*.

[41] Cuckle HS (2011). Screening for pre-eclampsia-lessons from aneuploidy screening. *Placenta*.

[42] Costa F, Murthi P, Keogh R, Woodrow N (2011). Early screening for preeclampsia. *Revista Brasileira de Ginecologia e Obstetrícia*.

[43] Pedrosa AC, Matias A (2011). Screening for pre-eclampsia: a systematic review of tests combining uterine artery Doppler with other markers.. *Journal of Perinatal Medicine*.

[44] Stepan H, Geipel A, Schwarz F, Krämer T, Wessel N, Faber R (2008). Circulatory soluble endoglin and its predictive value for preeclampsia in second-trimester pregnancies with abnormal uterine perfusion. *American Journal of Obstetrics and Gynecology*.

[45] Lockwood CJ, Krikun G, Caze R, Rahman M, Buchwalder LF, Schatz F (2008). Decidual cell-expressed tissue factor in human pregnancy and its involvement in hemostasis and preeclampsia-related angiogenesis. *Annals of the New York Academy of Sciences*.

[46] Schmidt M, Dogan C, Birdir C (2007). Altered angiogenesis in preeclampsia: evaluation of a new test system for measuring placental growth factor. *Clinical Chemistry and Laboratory Medicine*.

[47] Schmidt M, Dogan C, Birdir C (2009). Placental growth factor: a predictive marker for preeclampsia?. *Gynakologisch-geburtshilfliche Rundschau*.

[48] Akolekar R, Zaragoza E, Poon LCY, Pepes S, Nicolaides KH (2008). Maternal serum placental growth factor at 11 + 0 to 13 + 6 weeks of gestation in the prediction of pre-eclampsia. *Ultrasound in Obstetrics and Gynecology*.

[49] Vatten LJ, Eskild A, Nilsen TIL, Jeansson S, Jenum PA, Staff AC (2007). Changes in circulating level of angiogenic factors from the first to second trimester as predictors of preeclampsia. *American Journal of Obstetrics and Gynecology*.

[50] Rana S, Karumanchi SA, Levine RJ (2007). Sequential changes in antiangiogenic factors in early pregnancy and risk of developing preeclampsia. *Hypertension*.

[51] Kuc S, Wortelboer EJ, van Rijn BB, Franx A, Visser GHA, Schielen PCJI (2011). Evaluation of 7 serum biomarkers and uterine artery Doppler ultrasound for first-trimester prediction of preeclampsia: a systematic review. *Obstetrical and Gynecological Survey*.

[52] Mikat B, Zeller A, Scherag A (2012). *β*hCG and PAPP-A in first trimester: predictive factors for preeclampsia?. *Hypertens Pregnancy*.

[53] Spencer K, Cowans NJ, Nicolaides KH (2008). Low levels of maternal serum PAPP-A in the first trimester and the risk of pre-eclampsia. *Prenatal Diagnosis*.

[54] Akolekar R, Syngelaki A, Sarquis R, Zvanca M, Nicolaides KH (2011). Prediction of early, intermediate and late pre-eclampsia from maternal factors, biophysical and biochemical markers at 11–13 weeks. *Prenatal Diagnosis*.

[55] Akolekar R, Etchegaray A, Zhou Y, Maiz N, Nicolaides KH (2009). Maternal serum activin A at 11–13 weeks of gestation in hypertensive disorders of pregnancy. *Fetal Diagnosis and Therapy*.

[56] Akolekar R, Minekawa R, Veduta A, Romero XC, Nicolaides KH (2009). Maternal plasma inhibin A at 11–13 weeks of gestation in hypertensive disorders of pregnancy. *Prenatal Diagnosis*.

[57] Huppertz B, Sammar M, Chefetz I, Neumaier-Wagner P, Bartz C, Meiri H (2008). Longitudinal determination of serum placental protein 13 during development of preeclampsia. *Fetal Diagnosis and Therapy*.

[58] Khalil A, Cowans NJ, Spencer K, Goichman S, Meiri H, Harrington K (2009). First trimester maternal serum placental protein 13 for the prediction of pre-eclampsia in women with a priori high risk. *Prenatal Diagnosis*.

[59] Chafetz I, Kuhnreich I, Sammar M (2007). First-trimester placental protein 13 screening for preeclampsia and intrauterine growth restriction. *American Journal of Obstetrics and Gynecology*.

[60] Spencer K, Cowans NJ, Chefetz I, Tal J, Meiri H (2007). First-trimester maternal serum PP-13, PAPP-A and second-trimester uterine artery Doppler pulsatility index as markers of pre-eclampsia. *Ultrasound in Obstetrics and Gynecology*.

[61] Burger O, Pick E, Zwickel J (2004). Placental protein 13 (PP-13): effects on cultured trophoblasts, and its detection in human body fluids in normal and pathological pregnancies. *Placenta*.

[62] Garlanda C, Bottazzi B, Bastone A, Mantovani A (2005). Pentraxins at the crossroads between innate immunity, inflammation, matrix deposition, and female fertility. *Annual Review of Immunology*.

[63] Poon LCY, Akolekar R, Lachmann R, Beta J, Nicolaides KH (2010). Hypertensive disorders in pregnancy: screening by biophysical and biochemical markers at 11–13 weeks. *Ultrasound in Obstetrics and Gynecology*.

[64] Poon LCY, Stratieva V, Piras S, Piri S, Nicolaides KH (2010). Hypertensive disorders in pregnancy: combined screening by uterine artery Doppler, blood pressure and serum PAPP-A at 11–13 weeks. *Prenatal Diagnosis*.

[65] Granger JP, Alexander BT, Llinas MT, Bennett WA, Khalil RA (2001). Pathophysiology of hypertension during preeclampsia linking placental ischemia with endothelial dysfunction. *Hypertension*.

[66] Sifakis S, Akolekar R, Kappou D, Mantas N, Nicolaides KH (2011). Maternal serum insulin-like growth factor-binding protein-1 (IGFBP-1) at 11–13 weeks in pre-eclampsia. *Prenatal Diagnosis*.

[67] Sifakis S, Akolekar R, Kappou D, Mantas N, Nicolaides KH (2012). Maternal serum insulin-like growth factor-binding protein-3 (IGFBP-3) at 11–13 weeks in pre-eclampsia. *Journal of Human Hypertension*.

[68] Nanda S, Yu CKH, Giurcaneanu L, Akolekar R, Nicolaides KH (2011). Maternal serum adiponectin at 11–13 weeks of gestation in preeclampsia. *Fetal Diagnosis and Therapy*.

[69] Nanda S, Poon LCY, Muhaisen M, Acosta IC, Nicolaides KH (2012). Maternal serum resistin at 11 to 13 weeks’ gestation in normal and pathological pregnancies. *Metabolism*.

[70] Khalil AA, Tsikas D, Akolekar R, Jordan J, Nicolaides KH Asymmetric dimethylarginine, arfinine and homoarginine at 11–13 weeks gestation andpreeclampsia: a case-control study.

[71] Ives A, Saunders C, Bulsara M, Semmens J (2007). Pregnancy after breast cancer: population based study. *British Medical Journal*.

